# Bacterial Vaginosis, *Atopobium vaginae* and Nifuratel

**DOI:** 10.2174/157488412799218824

**Published:** 2012-02

**Authors:** Franco Polatti

**Affiliations:** Department of Obstetrics and Gynecology, Policlinico San Matteo, University of Pavia, Pavia, Italy

**Keywords:** Antibiotic resistance, *Atopobium vaginae*, bacterial vaginosis, nifuratel, review.

## Abstract

As bacterial vaginosis (BV) is a potential cause of obstetric complications and gynecological disorders, there is substantial interest in establishing the most effective treatment. Standard treatment - metronidazole or clindamycin, by either vaginal or oral route – is followed by relapses in about 30% of cases, within a month from treatment completion. This inability to prevent recurrences reflects our lack of knowledge on the origins of BV. *Atopobium vaginae *has been recently reported to be associated with BV in around 80% of the cases and might be involved in the therapeutic failures. This review looks at the potential benefits of nifuratel against *A. vaginae *compared to the standard treatments with metronidazole and clindamycin. *In vitro, *nifuratel is able to inhibit the growth of *A. vaginae*, with a MIC range of 0.125-1 µg/mL; it is active against *G. vaginalis *and does not affect lactobacilli. Metronidazole is active against *A. vaginae *only at very high concentrations (8-256 µg/mL); it is partially active against *G. vaginalis *and also has no effect on lactobacilli. Clindamycin acts against *A. vaginae *with an MIC lower than 0.125 µg/mL and is active on *G. vaginalis *but it also affects lactobacilli, altering the vaginal environment. These observations suggest that nifuratel is probably the most valid therapeutic agent for BV treatment.

## BACTERIAL VAGINOSIS 

### Epidemiology and Pathogenesis

Bacterial vaginosis (BV) is one of the most frequent female lower genital tract infections, not only in pregnancy but throughout the reproductive life. Studies from Europe and the USA have found prevalence between 4.9% and 36.0% [1]. The first signs of BV are radical changes in the vaginal ecosystem. H_2_0_2-_producing lactobacilli, which are present in 96% of women with normal vaginal bacterial flora, are markedly reduced or lost, while microorganisms like *Gardnerella vaginalis* and obligate anaerobes prevail [2]. The cause of this change is not clear [3] and the microorganisms responsible for the shift in the flora have still to be identified [4]. BV may be due not only to the excessive bacterial growth, but also to the formation of a dense bacterial biofilm adherent to the vaginal mucosa.

### Which is the Role of the Biofilm?

The biofilm formed by *Gardnerella* *vaginalis* in BV was first identified by electron microscopy as a dense tissue strongly adherent to the vaginal epithelium, and made up of bacterial cells packed inside a network of polysaccharide fibrils [5, 6]. Later, Swidsinki *et al*., investigating vaginal biopsies by bacterial rDNA fluorescent *in situ* hybridization, suggested that the bacterial biofilm played a primary role in the development of BV [7].

Costerton *et al*. and Swidsinki *et al*. found a dense bacterial biofilm, coating at least half the epithelial surface, in 90% of biopsies from women with BV, and in only 10% of healthy women [7, 8]. The presence of the biofilm enables the bacterial cells to reach higher concentrations (up to 10^11 ^bacteria/mL) than in vaginal fluid and boosts their resistance to both the host immune system and the antimicrobials [9, 10]. In fact, the drugs hardly reach the bacteria, residing inside the film in a quiescent state, leading to an up to 1000-fold antimicrobial decreased activity [9, 11]. This observation might provide an explanation of the high rates of BV relapses [10, 12]. 

### Complications of BV

**Fig. (1):**
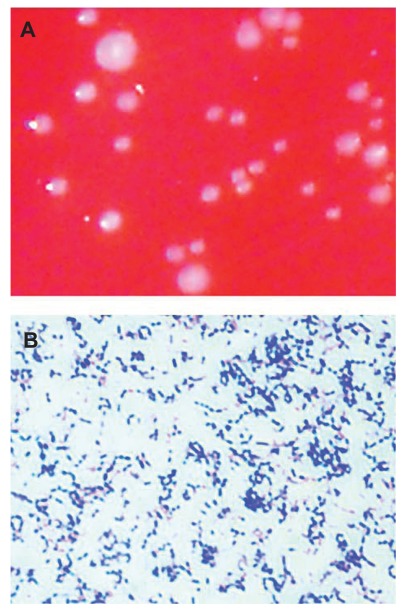
**A**) Grey-white colonies of *A. vaginae* after 48h culture in anaerobic conditions. **B**) Gram staining shows Gram-positive bacteria, with *A. vaginae* visible as single cells, in pairs or short chains. Geissdorfer *et al.* 2003 [41].

BV has aroused interest in the last few years being considered as a predisposing factor for HIV, Type II *Herpes symplex *virus, *Chlamydia trachomatis* infections, as well as for trichomoniasis and gonorrhea [13, 14]; BV can be also a cause for complications like late abortion [15], premature rupture of the amniotic membrane [16], chorio-amnionitis [17], *post-partum *endometritis [18, 19, 20], and failure of *in vitro *fertilization and embryo transfer [13, 14]. Particular attention has been recently paid to *Atopobium vaginae*, a newly identified bacterium, belonging to the *Coriobacteriaceae* family, which is believed to be at least a partial cause of the above mentioned complications [13]. The genus *Atopobium*, described for the first time in 1992, includes bacteria previously classified as lactobacilli. Rodriguez first identified *A. vaginae* in a study on vaginal lactobacilli [21]. *A. vaginae *16s rRNA gene differs from the other species belonging to *Atopobium* genus by approximately 3-8% [22, 23]; this enabled Rodriguez to identify it as a new species. The isolate can be distinguished from *A. minutum, A. parvulum, * and* A. rimae *by biochemical tests and protein electrophoresis of the whole cell (Table **1**). Gram stain shows *A. vaginae* as a small coccus, rounded or oval, or rods, visible as single cells, in pairs or in short chains (Fig. **1**).

**Fig. (2):**
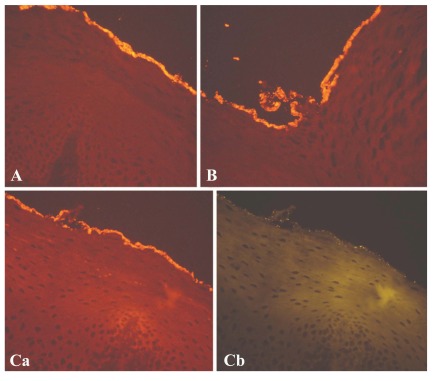
These microscopy images (**A**,**B**,**C**) show an unbroken *Gardnerella vaginalis* biofilm completely coating the vaginal epithelium. The lower panels show the same microscopic field (**Ca**) in dark-red fluorescence and (**Cb**) in orange fluorescence. Lactobacilli, interwoven with *G. vaginalis* in the film, only account for 5% of the bacterial population. Swidsinki *et al.* 2005 [7].

This aerobic facultative, gram-positive bacterium cannot be easily isolated by classical microbiological methods [14, 24]. It is hardly detected in healthy women vaginal fluid but is commonly found in the vagina of patients with BV: 50% according to Burton [25, 26], 70% according to Ferris [27], and more than 95% according to Verhelst *et al*. [24] and Verstraelen *et al*. [28]. In symptomatic BV it has been detected together with *Gardnerella vaginalis* in the biofilm adherent to the vaginal mucosa [24]. This was confirmed by Swidsinski *et al*. [7] who, by examining the composition and structural organization of the biofilm, found that Gardnerella vaginalis accounted for 60-95% of the film mass. In addition, in 70% of bioptic samples, *Atopobium vaginae* accounted for the 1-40% of the film mass. *Lactobacillus* concentrations were lower than 10^6^ CFU/mL, making up only 5% of the biofilm (Fig. **2**).

### Therapy 

Concerning the pharmacological therapy, CDC recommends either oral or topical (vaginal gel) metronidazole once a day for 5 days as first choice for BV. Efficacy is comparable to topical clindamycin [29]. Cure rates, following intravaginal treatment with metronidazole or clindamycin, account for 80-90% at the end of treatment and one month after the end of therapy [13, 14, 30]. However, three months after the end of therapy the rate of relapses can overcome 30%. Persistence of an adherent bacterial biofilm, containing mostly *G. vaginalis* and *A. vaginae*, seems to be the main reason for failure of BV treatment [30]. Suppressive treatment with metronidazole gel and physiological approaches (use of probiotics or acidifying) have been investigated with variable results [31]. Moreover, long-term treatment with metronidazole is not recommended because of the high incidence of gastrointestinal adverse reactions, the risk of peripheral neuropathy, and *Candida* super infection [32].

### Antibiotic Sensitivity

**Fig. (3):**
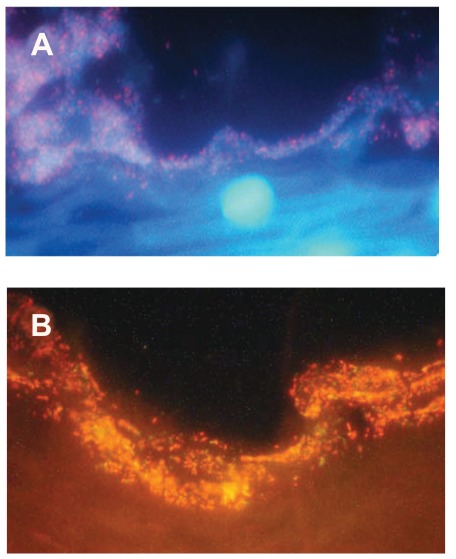
Microscopic images of the biofilm during and after treatment with metronidazole. **A**) Bacterial biofilm (x 400) in a patient at the third day of metronidazole therapy. The film is thin. **B**) Bacterial biofilm (x 400) in the same patient on day 35. The film has reformed almost completely. Swidsinki *et al.* 2008 [9].

Failures with metronidazole in patients with recurrent or persistent BV [33, 34] might conceivably reflect the newly found mechanism of formation of a biofilm containing *G. vaginalis* together with *A. vaginae* [7, 9, 13, 28] (Fig. **3**). The fact that *A. vaginae* is resistant to metronidazole, and that the bacterium creates a biofilm in which it is associated with *G. vaginalis,* complicates the response to the antibiotic [9, 13, 28]. Though clindamycin is more active than metronidazole against both *G. vaginalis* and *A. vaginae*, its negative effects on lactobacilli leave the way open to microbial disorders that can cause frequent super infections and recurrences. Moreover, an increasing resistance to antibiotics that act like clindamycin, by blocking protein synthesis has been reported. A randomized prospective trial compared 119 women assigned to two therapeutic regimens for BV: either metronidazole vaginal gel for five days, or clindamycin vaginal tablets for three days. The clinical efficacy was comparable in the two arms: after 7-12 days about 80% of the patients were cured, but this percentage fell down to about 50% after 35-45 days. Following clindamycin treatment – but not metronidazole - there was a steep rise in the percentage of women with at least one clindamycin resistant strain isolated. Moreover, 70-90 days after the end of treatment, about 80% of the women who received clindamycin presented in their vaginal swabs anaerobic bacteria resistant to that drug [35].

Togni *et al*. [36] compared the *in vitro* susceptibility of A. *vaginae* to nifuratel, metronidazole and clindamycin. Susceptibility to metronidazole was variable, with MIC ranging from 8 to 256 µg/mL. Nifuratel and clindamycin inhibited the growth of all the tested strains, with MIC from 0.125 to 1 µg/mL and below 0.125 µg/mL, respectively (Table **2**). The findings related to metronidazole and clindamycin are in line with previously published studies [37]. 

**Fig. (4):**
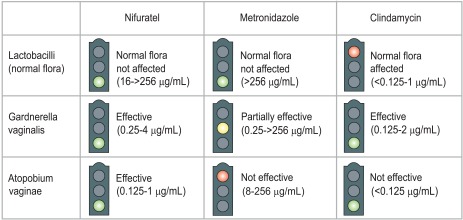
Activity of nifuratel, metronidazole and clindamycin on lactobacilli, *Gardnerella vaginalis* and *Atopobium vaginae.*

In the same study, the activity of these antibiotics was assayed on lactobacilli and *G. vaginalis*. Either nifuratel and metronidazole did not affect the normal lactobacterial flora, while clindamycin inhibited all tested strains of lactobacilli. Nifuratel and metronidazole were both highly active against *G. vaginalis* (Fig. **4**). The susceptibility of *Atopobium vaginae* to metronidazole and clindamycin, and the action on lactobacilli and *G. vaginalis* were in line with previous reports [37-39]. To summarise, nifuratel was active against *A. vaginae *and *G. vaginalis *strains without affecting lactobacilli; metronidazole was active against *A. vaginae*, but only at very high concentrations, partially active against *G. vaginalis,* and did not affect lactobacilli; clindamycin was extremely effective against *A. vaginae *and *G. vaginalis,* but it also affected the lactobacilli, altering the vaginal ecosystem.

## CONCLUSIONS

The discovery of the presence of *Atopobium vaginae* in the vaginal ecosystem improves the basic understanding of the pathogenesis of BV [28]. This bacterium is presumably the main reason for failures or recurrences after BV treatment with metronidazole, since it is found in 80-90% of cases of relapse [40]. Prospective studies are now needed to show whether metronidazole–resistant microorganisms, such as *Atopobium vaginae,* are involved in recurrences. Information to date suggests that nifuratel is probably the most valid therapeutic agent for BV, as it is highly active against *Gardnerella vaginalis *and *Atopobium vaginae, *without affecting lactobacilli which are fundamental for the system health and balance [30].

## Figures and Tables

**Table 1.** Biochemical Tests to Distinguish *A. vaginae* from the other *Atopobium* Species

Enzyme	*A. vaginae*	*A. minutum*	*A. parvulum*	*A. rimae*
Acid phosphatase	+	-	+	+
Alanine arylamidase	-	-	+	-
Arginine dihydrolase	+	+	-	-
Arginine arylamidase	+	+	+	-
Histidine arylamidase	+	-	-	-
B-Galactosidase	-	-	+	-
Leucine arylamidase	+	+	-	-
Proline arylamidase	+	+	-	-
Pyroglutamic acid arylamidase	-	v	+	+
Glycine arylamidase	+	-	+	-
Serine arylamidase	+	-	-	-
Thyroxine arylamidase	-	-	+	-

+, the enzyme is expressed constitutively; -, the enzyme is absent and cannot be induced; v, expression of the enzyme is variable Modified, from Rodriguez et al. 1999 [21].

**Table 2.** MIC Ranges (µg/mL) and MIC_50_ (µg/mL) of Metronidazole, Clindamycin and Nifuratel against *Atopobium vaginae*

Antimicrobial Agent	MIC Range (µg/ml)	MIC_50_ (µg/ml)
Metronidazole	8 - 256	32
Clindamycin	< 0.125	< 0.125
Nifuratel	0.125 - 1	0.5

Togni *et al.* 2011 [30].

## References

[1]  Morris M, Nicoll A, Simms I, Wilson J, Catchpole M (2001). Bacterial
vaginosis: a public health review. BJOG.

[2]  Nam H, Whang K, Lee Y (2007). Analysis of vaginal lactic acid producing
bacteria in healthy women. J Microbiol.

[3]  Cauci S, Monte R, Driussi S, Lanzafame P, Quadrifoglio F (1998). Impairment of the mucosal immune system: IgA and IgM cleavage
detected in vaginal washing of a subgroup of patients with bacterial
vaginosis. J Infect Dis.

[4]  Sobel JD (2000). Bacterial vaginosis. Annu Rev Med.

[5]  van der Meijden WI, Koerten H, van Mourik W, de Bruijn WC (1988). Descriptive light and electron microscopy of normal and clue-cell-positive
discharge. Gynecol Obstet Invest.

[6]  Scott TG, Curran B, Smyth CJ (1989). Electron microscopy of adhesive
interactions between *Gardnerella vaginalis* and vaginal epithelial
cells, McCoy cells and human red blood cells. J Gen Microbiol.

[7]  Swidsinki A, Mendling W, Loening-Baucke V, Ladhoff A, Swidsinki S, Hale LP, Lochs H (2005). Adherent biofilms in bacterial
vaginosis. Obstet Gynecol.

[8]  Costerton W, Veeh R, Shirtliff M, Pasmore M, Post C, Ehrlich G (2003). The application of biofilm science to the study and control of
chronic bacterial infections. J Clin Invest.

[9]  Swidsinki A, Mendling W, Loening-Baucke V, Swidsinski S, Dörffel Y, Scholze J, Lochs H, Verstraelen H (2008). An adherent
*Gardnerella vaginalis* biofilm persists on the vaginal epithelium
after standard therapy with metronidazole. Am J Obstet Gynecol.

[10]  Hay P (2000). Recurrent bacterial vaginosis. Curr Infect Dis Rep.

[11]  Hoiby N, Bjarnsholt T, Givskov M, Molin S, Ciofu O (2010). Antibiotic
resistance of bacterial biofilms. Int J Antimicrob Agents.

[12]  Pirotta M, Fethers KA, Bradshaw CS (2009). Bacterial vaginosis - More
questions than answers. Aust Fam Physician.

[13]  Fredricks DN, Fiedler TL, Marrazzo JM (2005). Molecular identification
of bacteria associated with bacterial vaginosis. N Engl J Med.

[14]  Livengood CH (2009). Bacterial vaginosis: an overview for 2009. Rev Obstet Gynecol.

[15]  Donati L, Di Vico A, Nucci M, Quagliozzi L, Spagnuolo T, Labianca A, Bracaglia M, Ianniello F, Caruso A, Paradisi G (2010). Vaginal microbial flora and outcome of pregnancy. Arch Gynecol
Obstet.

[16]  McDonald HM, Brocklehurst P, Gordon A (2007). Antibiotics for treating
bacterial vaginosis in pregnancy. Cochrane Database Syst Rev.

[17]  Fahey JO (2008). Clinical management of intra-amniotic infection and
chorioamnionitis: a review of the literature. J Midwifery Women’s
Health.

[18]  Hillier SL, Kiviat NB, Hawes SE, Hasselquist MB, Hanssen PW, Eschenbach DA, Holmes KK (1996). Role of bacterial vaginosis-associated
microorganisms in endometritis. Am J Obstet Gynecol.

[19]  Sweet RL (1995). Role of bacterial vaginosis in pelvic inflammatory
disease. Clin Infect Dis.

[20]  Pellati D, Mylonakis I, Bertoloni G, Fiore C, Andrisani A, Ambrosini G, Armanini D (2008). Genital tract infections and infertility. Eur J Obstet
Gynecol Reprod Biol.

[21]  Rodriguez Jovita M, Collins MD, Sjoden B, Falsen E (1999). Characterization
of a novel *Atopobium* isolate from the human vagina: description
of *Atopobium vaginae* sp. nov. Int J Syst Bacteriol.

[22]  Collins MD, Wallbanks S (1992). Comparative sequence analysis of the
16s rRNA genes of *Lactobacillus minutus, Lactobacillus rimae* and
*Streptococcus parvulus:* proposal for the creation of a new genus
Atopobium. FEMS Microbiol Lett.

[23]  Stackebrandt E, Ludwig W (1994). The importance of using outgroup
reference organisms in phylogenetic studies: the *Atopobium* case. Syst Appl Microbiol.

[24]  Verhelst R, Vestraelen H, Claeys G, Verschraegen G, Delanghe J, Van Simaey L, De Ganck C, Temmerman M, Vaneechoutte M (2004). Cloning of 16S rRNA genes amplified from normal and
disturbed vaginal microflora suggests a strong association between
*Atopobium vaginae, Gardnerella vaginalis* and bacterial vaginosis. BMC Microbiol.

[25]  Burton JP, Devillard E, Cadieux PA, Hammond JA, Reid G (2004). Detection
of *Atopobium vaginae* in postmenopausal women: cultivation-independent
methods warrants further investigation. J Clin Microbiol.

[26]  Burton JP, Chilcott CN, Al-Qumber M, Brooks HJ, Wilson D, Tagg JR, Devenish C (2005). A preliminary survey of *Atopobium vaginae*
in women attending the Dunedin gynaecology out-patients clinic: is
the contribution of the hard-to-culture microbiota overlooked in
gynaecological disorders?. Aust N Z J Obstet Gynaecol.

[27]  Ferris MJ, Masztal A, Martin DH (2004). Use of species-directed 16S rRNA
gene PCR primers for detection of *Atopobium vaginae* in patients
with bacterial vaginosis. J Clin Microbiol.

[28]  Vestraelen H, Verhelst R, Claeys G, Temmerman M, Vaneechoutte M (2004). Culture-independent analysis of vaginal microflora: the unrecognized
association of *Atopobium vaginae* with bacterial vaginosis. Am J Obstet Gynecol.

[29]  Workwoski KA, Berman S (2010). Centers for Disease Control and
Prevention. Sexually transmitted disease treatment guidelines 2010. Recommandation and Reports.

[30]  Togni G, Battini V, Bulgheroni A, Mailland F, Caserini M, Mendling W (2011). *In vitro* activity of nifuratel on vaginal bacteria:
could it be a good candidate for the treatment of bacterial vaginosis?. Antimicrob Agents Chemother.

[31]  Hay P (2009). Recurrent bacterial vaginosis. Current Opinion in infectious
diseases.

[32]  Dickey LJ, Nailor MD, Sobel JD (2009). Guidelines for the treatment of
bacterial vaginosis: focus on tinidazole. Ther Clin Risk Management.

[33]  Ferris DG, Litaker MS, Woodward L, Mathis D, Hendrich J (1995). Treatment of bacterial vaginosis: a comparison of oral metronidazole,
metronidazole vaginal gel and clindamycin vaginal cream. J
Fam Pract.

[34]  Larsson PG, Forsum U (2005). Bacterial vaginosis: a disturbed bacterial
flora and treatment enigma. APMIS.

[35]  Fredricks DN, Fiedler TL, Thomas KK, Mitchell CM, Marrazzo JM (2009). Changes in vaginal bacterial concentrations with intravaginal
metronidazole therapy for bacterial vaginosis as assessed by quantitative
PCR. J Clin Microbiol.

[36]  Beigi RH, Austin MN, Meyn LA, Krohn MA, Hillier SL (2004). Antimicrobial
resistance associated with the treatment of bacterial vaginosis. Am J Obstet Gynecol.

[37]  De Backer E, Verhelst R, Vestraelen H (2006). Antibiotic susceptibility
of *Atopobium vaginae*. BMC Infect Dis.

[38]  Goldstein EJ, Citron DM, Merriam CV, Warren YA, Tyrrell KL, Fernandez HT (2002). *In vitro* activities of Garenoxacin (BMS 284756)
against 108 clinical isolates of *Gardnerella vaginalis*. Antimicrob
Agents Chemother.

[39]  Nagaraja P (2008). Antibiotic resistance of *Gardnerella vaginalis* in recurrent
bacterial vaginosis. Indian J Med Microbiol.

[40] Hillier SL, Homes KK,  Homes KK, Sparling PF, Mardh PA, Lemon SM, Stamm WE, Piot P, Wasserheit (1999). Bacterial vaginosis, in Sexually Transmitted
Diseases.

[41]  Geissdorfer W, Bohmer C, Pelz K, Schoerner C, Frobenius W, Bogdan C (2003). Tubo-ovarian abscess caused by *Atopobium vaginae*
following transvaginal oocyte recovery. J Clin Microbiol.

